# Molecularly Imprinted Polymer Biosensors for Sustainable Antiretroviral Drug Monitoring

**DOI:** 10.3390/s26216926

**Published:** 2026-11-02

**Authors:** Christopher Animashaun, Gymama Slaughter

**Affiliations:** 1 Center for Bioelectronics, Old Dominion University, Norfolk, VA 23508, USA; 2 Department of Electrical and Computer Engineering, Old Dominion University, Norfolk, VA 23508, USA

**Keywords:** molecularly imprinted polymers, antiretroviral drug monitoring, electrochemical biosensors, point-of-care analysis, green analytical chemistry

## Abstract

Antiretroviral therapy (ART) and pre-exposure prophylaxis (PrEP) have transformed human immunodeficiency virus (HIV) treatment and prevention, but both depend on sustained adherence and accurate drug-exposure monitoring. Liquid chromatography-tandem mass spectrometry remains the reference method for antiretroviral quantification, but centralized instrumentation and multistep sample preparation limit decentralized, same-visit testing. This review evaluates molecularly imprinted polymers (MIPs) as synthetic recognition elements for optical and electrochemical antiretroviral sensing, situated within the broader case for green analytical chemistry in decentralized HIV care. MIP-based platforms are compared with direct electrochemical detection and aptamer-based sensing across selectivity, stability, fabrication complexity, regeneration, and matrix tolerance in plasma, dried blood spots, urine, saliva, and interstitial fluid. Although recent MIP sensors achieve nanomolar detection, none of the reviewed studies report standardized greenness metrics such as AGREE, GAPI, or the Analytical Eco-Scale, limiting comparison of their true environmental performance. Clinical value remains further constrained by limited validation in authentic specimens, nonspecific adsorption, and batch-to-batch variability. Future progress will depend on simpler MIP architectures, safer extraction chemistries, matrix-matched validation, and systematic sustainability assessment using established green analytical chemistry metrics.

## 1. Background

### 1.1. Clinical Rationale for Antiretroviral Drug Monitoring

Antiretroviral therapy (ART) has transformed HIV infection into a manageable chronic condition, while pre-exposure prophylaxis (PrEP) has expanded prevention options [1,2,3]. Both treatment and prevention depend on sustained drug exposure, yet adherence is difficult to assess reliably using self-report, pill counts, or pharmacy records alone [4,5,6,7]. Objective drug-level measurements can therefore complement behavioral adherence measures and help distinguish recent dosing from cumulative exposure. Therapeutic drug monitoring (TDM) measures drug concentrations to support dose optimization, evaluate suspected non-adherence, identify drug–drug interactions, and reduce the risk of toxicity or treatment failure [8,9,10,11]. In HIV care, drug-level data are complementary to viral-load testing because viral load indicates whether treatment is working, whereas antiretroviral concentrations can help explain why treatment may be failing. Subtherapeutic exposure may result from missed doses, altered absorption or clearance, or drug–drug interactions. When drug concentrations remain inadequate, viral replication and resistance can emerge [12,13]. Additionally, long-acting agents such as cabotegravir and lenacapavir reduce daily pill burden, but they create new monitoring needs because drug exposure persists over extended dosing intervals [14,15]. Together, these dynamics support the need for analytical methods that are sensitive enough for relevant concentration ranges but also sufficiently rapid, accessible, and reliable for decentralized care.

The evolving landscape of antiretroviral treatment and prevention further highlights the need for drug-specific monitoring strategies. Dolutegravir has been extensively investigated through pharmacokinetic analyses of plasma and urine concentrations [16], while long-acting cabotegravir and emerging extended-interval lenacapavir regimens introduce additional considerations related to prolonged drug exposure and dosing intervals [14,15,17]. These developments expand the analytical scope beyond commonly investigated oral antiretrovirals, such as tenofovir and emtricitabine, and highlight the importance of distinguishing between parent-drug concentrations in plasma, urinary markers of recent dosing, and biomarkers reflecting longer-term exposure. Because these measurements represent different pharmacokinetic processes and adherence windows, their concentration ranges and clinical interpretations are not directly interchangeable. Collectively, these considerations identify opportunities for developing MIP-based sensing platforms tailored to specific antiretroviral drugs, biological matrices, and clinically relevant monitoring objectives.

### 1.2. Limitations of Conventional Analytical Methods

Liquid chromatography-tandem mass spectrometry (LC-MS/MS) remains the reference method for antiretroviral quantification because it provides high sensitivity, selectivity, and multiplexing capability when supported by validated sample preparation, internal standards, and matrix-effect assessment [18]. Its limitations for decentralized care arise from instrument cost, specialized personnel, multistep preparation, and dependence on centralized laboratory workflows. High-performance liquid chromatography with ultraviolet or fluorescence detection can reduce instrument complexity relative to LC-MS/MS, but it still requires trained personnel, sample preparation, and laboratory infrastructure, and it may provide lower selectivity in complex matrices [19,20,21]. These constraints limit the value of conventional methods for same-visit adherence assessment in settings where samples must be transported to centralized laboratories. The need for rapid, portable, and lower-resource testing is especially important in decentralized and resource-limited settings, where transport, storage, batching, and laboratory turnaround can delay clinical decisions [22,23,24]. Biosensors are therefore best viewed as potential complements to reference methods rather than automatic replacements.

### 1.3. Biosensing Rationale, Targets, and Sample Matrices

**Figure 1:**
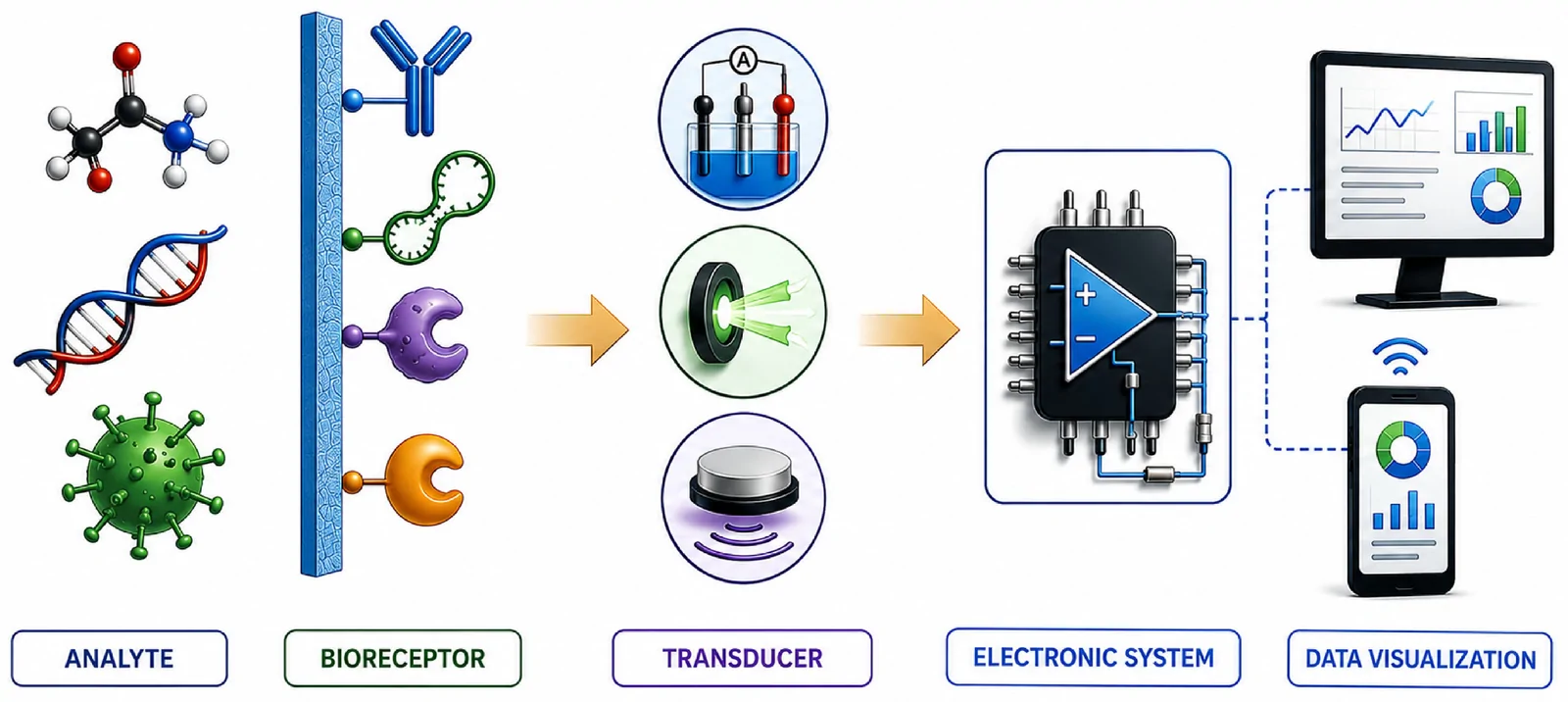
General architecture of a biosensor. Target analytes, including small molecules, DNA, and viruses, are selectively recognized by a bioreceptor such as an antibody, aptamer, receptor, or enzyme. The biorecognition event is converted into a measurable signal by an electrochemical, optical, or piezoelectric transducer, followed by signal processing and visualization through the electronic system.

Biosensors couple a recognition interface with a transducer to convert target binding or electrochemical reaction into a measurable signal [25,26]. For antiretroviral monitoring, the central design question is whether this signal can be linked reproducibly to clinically relevant drug concentrations in the intended sample matrix. The recognition interface in Figure 1 determines what is detected; the transducer determines how that event becomes a readable output. Recognition elements range from biological receptors, such as enzymes and antibodies, to synthetic alternatives, including aptamers and molecularly imprinted polymers (MIPs), while transducers report binding through electrochemical, optical, or mass-sensitive signals. Electrochemical formats are generally more compatible with low-cost, portable instrumentation than optical or mass-sensitive alternatives. A further consideration is whether detection is direct or requires a labeled reporter: label-free formats reduce reagent consumption, relevant to green chemistry, while labeled formats can improve sensitivity at the cost of added handling steps that work against decentralized, same-visit use.

The selection of a recognition interface is particularly important for antiretroviral drug monitoring because the targeted compounds differ in molecular structure, physicochemical properties, therapeutic concentration ranges, and biological distribution. Although biological recognition elements have established the foundation for selective biosensing, their susceptibility to environmental conditions and degradation can complicate device storage and deployment [27,28]. Molecularly imprinted polymers offer a synthetic alternative through polymeric binding sites designed to recognize specific target molecules, with potential advantages in physicochemical stability, fabrication flexibility, and reduced reliance on biological reagents. However, these advantages do not inherently guarantee superior analytical performance or environmental sustainability. The suitability of MIP-based sensors must instead be established through evidence of target selectivity, binding-site accessibility, signal reproducibility, matrix tolerance, and the resource demands associated with polymer synthesis, template removal, and sensor fabrication.

None of these choices can be evaluated in isolation from the sample matrix, since a response measured in buffer does not establish that it will hold in plasma, urine, or saliva, where competing analytes and nonspecific binding can distort the signal. Answering the central design question therefore depends on how recognition chemistry and transducer are validated together, in the matrix the sensor is ultimately meant to test.

Electrochemical and optical paper-based aptamer and molecularly imprinted platforms provide useful benchmarks for evaluating the distinctive advantages and limitations of MIPs in antiretroviral sensing. Limits of detection (LOD) and linear range (LR) are useful analytical descriptors, but they are insufficient for assessing clinical or environmental value. Matrix tolerance, selectivity against related drugs and endogenous interferents, batch reproducibility, calibration transfer, sample preparation, storage stability, regeneration, and agreement with a reference method are equally important. Antiretroviral sensing studies have focused mainly on tenofovir, emtricitabine, nevirapine, and efavirenz, with much less coverage of integrase inhibitors and long-acting agents [29,30,31,32,33,34,35]. Parent-drug and metabolite measurements answer different adherence questions, and the selected matrix determines the time window represented by the result [16,34,35]. Moreover, blood, plasma, dried blood spots, urine, saliva, and interstitial fluid each offer different balances of invasiveness, analyte abundance, stability, and sample-processing requirements [36,37,38,39,40,41,42]. Urine is attractive for recent tenofovir exposure, dried blood spots can reflect cumulative intracellular metabolite exposure, and saliva or interstitial fluid may support less invasive monitoring, but matrix-specific calibration and validation are essential.

This review focuses on MIPs as smart synthetic recognition components for antiretroviral drug monitoring. Conventional analytical methods and alternative biosensor classes are discussed primarily as benchmarks for evaluating the analytical and translational value of MIP-based platforms. The review examines how template chemistry, functional monomer selection, polymer thickness, extraction conditions, electrode architecture, and sample matrix jointly shape sensor performance. It also critically assesses whether reported systems advance sustainable analysis through reduced sample volume, lower reagent and energy requirements, reusable recognition interfaces, and compatibility with decentralized point-of-care testing, to deliver reproducible, clinically meaningful, and environmentally responsible measurements in authentic samples. This dual framing follows the White Analytical Chemistry perspective, which holds that analytical methods should be evaluated jointly on environmental impact, practical applicability, and analytical performance rather than on greenness in isolation [43].

## 2. Electrochemical Transducers for Molecularly Imprinted Antiretroviral Sensors

### 2.1. Why Electrochemical Sensing Is Attractive

Electrochemical transducers are attractive for MIP-based drug sensing because they can be miniaturized, coupled to portable potentiostats, and operated with small sample volumes [23,44]. These features may reduce energy demand and support same-visit testing. However, a portable format is not, by itself, a clinical or environmental credential. Device performance must also be evaluated against matrix effects, calibration requirements, disposable material use, and the environmental burden of electrode production and template extraction [44,45]. Electrode material and nanostructuring strategy are central to this evaluation, since both shape the achievable sensitivity and the resource footprint of fabrication. Carbon electrodes, laser-scribed graphene, gold, conductive polymers, metal nanoparticles, and metal–organic frameworks have been used to improve surface area, conductivity, and signal amplification [46,47,48,49,50]. Isolating their contribution from that of molecular imprinting requires care, since a hybrid platform may produce a lower detection limit because of the transducer architecture rather than improved molecular recognition. Therefore, studies typically include an uncoated transducer and/or a non-imprinted polymer control.

### 2.2. Recognition Strategies and the Role of Molecular Imprinting

Direct electrooxidation, aptamer functionalization, and molecular imprinting provide different routes to antiretroviral quantification. Each strategy trades off selectivity, stability, and fabrication complexity differently, so the appropriate choice depends on the specific drug target and intended testing context rather than any single platform being universally superior. Direct electrooxidation offers rapid and comparatively simple detection when the drug is electroactive, but its selectivity can be limited by overlapping responses from endogenous compounds [51]. Aptamers provide sequence-programmable affinity, but small-molecule target recognition can be sensitive to immobilization, folding, and selection conditions [52]. MIPs replace biological receptors with synthetic binding cavities and therefore offer greater chemical and storage stability [49]. Their performance, however, depends strongly on monomer–template interactions, film thickness, extraction efficiency, and the accessibility and uniformity of the imprinted sites. These dependencies mean that MIP performance is largely fabrication-defined rather than intrinsic to the recognition chemistry itself, in contrast to the comparatively fixed binding behavior of a validated aptamer sequence or the direct dependence of electrooxidation on the drug’s own electroactivity.

**Figure 2:**
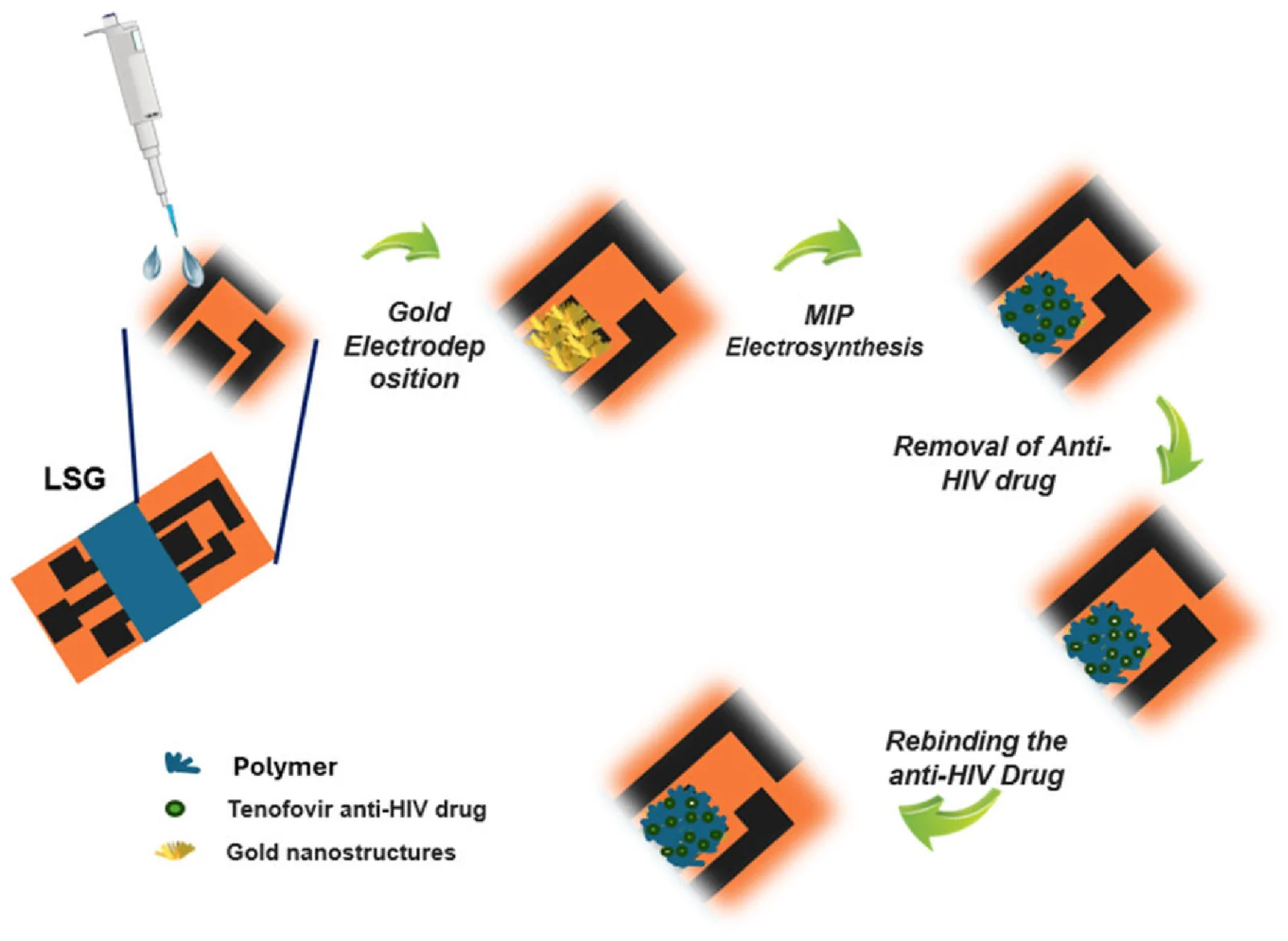
Schematic illustration of LSG/AuNSp/MIP biomimetic sensor preparation and surface modification. Adapted from [53].

Representative tenofovir sensors depicted in Table 1 illustrate the trade-off between analytical performance and platform complexity. Lahcen et al. reported an LSG/AuNSp/MIP platform with a 10 nM to 200 µM range and a 3 nM detection limit [53]. Figure 2 depicts the fabrication sequence underlying this platform. Gold is first electrodeposited onto the laser-scribed graphene surface to form spiky gold nanostructures, which increase effective surface area and provide sites for subsequent polymer growth. A molecularly imprinted layer is then electrosynthesized around the tenofovir template directly on this gold-modified surface, so the imprinted cavities form in immediate contact with the conductive nanostructures rather than within a separately blended composite. Template removal extracts the tenofovir to leave complementary binding cavities, and rebinding of the analyte during the assay regenerates the interaction that generates the measured signal. Because gold electrodeposition, imprinting, extraction, and rebinding occur in direct succession on the same surface, inconsistency introduced at any step, for example, uneven gold nanostructure formation, can propagate through polymer growth and affect the reproducibility of the final imprinted cavities. Mehmandoust et al. reported a multilayer MIP-Pt@g-C_3_N_4_/F-MWCNT/SPE platform with a 0.005 to 0.69 µM range and a 3 nM detection limit [54]. Both approaches demonstrate nanomolar detection, but the latter requires multiple material layers and processing steps. A comparatively contained architecture, comprising one nanostructuring step followed by one imprinting step, is consistent with why the LSG/AuNSp/MIP platform achieves comparable detection at only “moderate” fabrication difficulty in Table 1, compared with the MIP-Pt@g-C_3_N_4_/F-MWCNT/SPE “difficult (multiple modifications)” rating. Such complexity may improve signal amplitude while increasing cost, material use, and batch variation.

The analytical performance demonstrated for tenofovir and emtricitabine cannot be assumed to extend directly to other antiretroviral drugs, including the integrase strand transfer inhibitors dolutegravir and bictegravir, or the long-acting agents cabotegravir and lenacapavir [33,53]. Differences in molecular structure, solubility, functional-group availability, and electrochemical behavior may influence template–monomer interactions, imprint formation, analyte rebinding, and signal transduction. Extending MIP-based sensing to these drugs therefore requires drug-specific evaluation of imprinting chemistry, recognition-site accessibility, matrix compatibility, and clinically relevant concentration ranges. Redox-probe-based detection and surface-confined mediators represent potential transduction strategies, but their suitability must be established experimentally because effective electron transfer does not necessarily indicate selective analyte recognition. Consequently, the low detection limits achieved with existing antiretroviral MIP sensors provide a foundation for further development rather than direct evidence of transferable analytical performance.

Beyond electrode nanostructuring, the spatial distribution and accessibility of imprinted recognition sites are important determinants of MIP sensor performance. The tenofovir LSG/AuNSp/MIP and emtricitabine LIG/PB/MIP platforms incorporate imprinted polymer layers directly onto conductive electrode architectures [33,53], while the nevirapine MIP/ErGO system illustrates the integration of molecular imprinting with a carbon-based electrode modification [55]. These configurations highlight the importance of controlling polymer thickness, binding-site accessibility, and analyte transport between the sample solution and the electrode interface. Surface-oriented imprinting strategies may reduce diffusion distances and improve binding and rebinding kinetics by positioning recognition sites closer to the polymer–solution interface. However, the contribution of imprint location cannot be established from detection limits alone, particularly when changes in electrode surface area, conductivity, polymer morphology, or redox mediation also influence the measured response. Demonstrating a specific advantage therefore requires appropriately matched controls, characterization of polymer structure and thickness, and comparative evaluation of binding kinetics and analytical performance.

Emtricitabine has also been addressed using a reagent-free approach. Lahcen et al. reported an LIG/PB/MIP platform in which an electrodeposited Prussian Blue layer serves as an internal redox mediator, eliminating the need for an external ferri/ferrocyanide probe, with an FTC-imprinted polypyrrole film electropolymerized directly on the Prussian Blue surface [33]. This sensor achieved a 10 nM to 200 µM linear range and a 5 nM detection limit. Notably, this study addressed several of the evidentiary gaps: performance was benchmarked against a non-imprinted control (imprinting factor of 2.54), cross-reactivity was tested against tenofovir and lamivudine alongside common electroactive interferents, and the sensor was evaluated in both synthetic urine and protein-enriched synthetic urine rather than buffer alone. Reusability, however, was limited to three rebinding cycles before regeneration efficiency declined, and storage stability, while favorable through two weeks, showed increasing sensor-to-sensor variability by day 20, limitations the study identifies as requiring further characterization before clinical translation.

For nevirapine, reported platforms combine metal–organic frameworks, MWCNTs, MXenes, reduced graphene oxide, or imprinted polymers [55,56]. Reported nanomolar detection and recovery in prepared matrices are encouraging, but recovery figures say little about selectivity on their own. More informative evidence would include cross-reactivity with related antiretrovirals, independent-batch reproducibility, authentic-specimen testing, and agreement with LC-MS/MS. For efavirenz, reported electrochemical platforms span a reduced graphene oxide-platinum composite [57], a nickel oxide-zirconium dioxide nanocomposite [58], and an unmodified pencil graphite electrode [59], none of which incorporate molecular imprinting. These platforms report detection limits in the low-nanomolar range and have been validated in urine, plasma, and tablet formulations, but as with the nevirapine platforms above, reported recovery and detection-limit data do not by themselves establish selectivity against structurally related antiretrovirals or agreement with a reference method in authentic clinical matrices.

Considered together, several non-imprinted comparator platforms, including the SAM/MCH aptasensor [52], the BZK/AgNPs sensor [62], and the pencil graphite electrode [61], achieve detection limits comparable to or lower than the MIP-based platforms [53,54,55], despite requiring fewer fabrication steps. This does not diminish the case for molecular imprinting, but it does mean that detection limit alone cannot justify the added fabrication and extraction burden that imprinting introduces; the case for MIPs has to rest on selectivity, reusability, and storage stability rather than on sensitivity competing head-to-head with simpler electrode modifications. Beyond these individual platforms, the broader pattern across Table 1 is that low detection limits are commonly achieved through increasingly complex hybrid architectures that combine carbon nanomaterials, noble metals, metal–organic frameworks, or MIPs. These additions can improve surface area and electron transfer, but they also increase the number of fabrication steps, material inputs, and potential sources of batch variation. Consequently, the lowest reported detection limit should not be treated as the sole indicator of platform quality. For point-of-care use, analytical sensitivity must be considered together with fabrication reproducibility, sample preparation, matrix tolerance, device cost, and the environmental burden of synthesis and disposal.

MIP-containing systems consistently reach nanomolar detection while providing synthetic recognition without the storage limitations of biological receptors [53,54,55]. Their main advantage is, however, not necessarily superior sensitivity, since several non-imprinted electrodes achieve comparable limits of detection, but rather the combination of selectivity, chemical robustness, and potential reusability. The principal trade-off is that multilayer MIP nanocomposites may require complex synthesis and template-removal procedures that weaken their suitability for scalable and sustainable production. Fabrication complexity is not the only barrier to deployment, however: clinical validation remains the most important gap. Most studies rely on synthetic urine, pharmaceutical formulations, spiked matrices, or a limited number of extracted biological samples. These tests do not fully reproduce the ionic strength, proteins, metabolites, and patient-to-patient variability encountered in authentic specimens. Claims of clinical readiness therefore warrant caution until MIP sensors are compared directly with validated reference methods using adequately powered clinical sample sets.

The literature is also concentrated on tenofovir, with fewer studies addressing nevirapine, efavirenz, integrase inhibitors, or long-acting antiretroviral agents. This imbalance limits the relevance of current MIP platforms to contemporary treatment regimens. Expanding imprinting strategies to newer therapies will require target-specific assessment of functional monomers, extraction solvents, cross-reactivity, and clinically meaningful concentration ranges rather than simple transfer of established tenofovir designs. Overall, electrode engineering and molecular imprinting have improved the analytical capabilities of antiretroviral sensors, but successful translation will depend on simplifying architectures, reducing material and solvent use, improving batch reproducibility, validating performance in authentic specimens, and demonstrating that the selected design provides a clinically useful advantage over simpler electrochemical or affinity-based alternatives.

## 3. Aptamer-Based Biosensors as Biological Comparators

### 3.1. Analytical Strengths of Aptamers

Aptamers provide sequence-defined recognition and can be integrated with electrochemical and optical transducers [63,64,65]. Their principal advantage over MIPs is potentially more uniform binding chemistry when a validated sequence is available, since a single characterized oligonucleotide sequence reproduces the same binding pocket in every batch, whereas MIP cavity formation depends on stochastic polymer growth around the template. Aptamers are also chemically synthesized rather than biologically expressed, which avoids the animal-derived production and lot-to-lot variability associated with antibodies [66]. Their limitations include the cost and time of selection, sensitivity to immobilization density and folding conditions, and uncertain affinity for some small-molecule drugs.

Small antiretroviral molecules provide limited binding surfaces, which can make aptamer selection and discrimination from structurally related compounds difficult [67,68,69]. This is a structural limitation rather than a procedural one: SELEX depends on selecting sequences that fold around a target with enough surface contact to generate high-affinity, high-specificity binding, and small molecules simply present fewer distinguishing chemical features than the proteins aptamers were originally developed against [68,70]. The SELEX process iteratively partitions bound from unbound sequences across a random oligonucleotide library and amplifies the enriched pool; for small-molecule targets, this partitioning step is technically harder than for protein targets because the free small molecule and the aptamer-bound complex are closer in size and behavior, making efficient physical separation between rounds more difficult and potentially prolonging selection. Antiretroviral drugs that differ only by a single functional group, as within a drug class, can therefore be difficult to discriminate using aptamers selected against the parent structure. Surface immobilization can further alter aptamer conformation and reduce accessible binding sites. These constraints therefore shape how aptamers are used as benchmarks for MIP performance.

Beyond their use as independent recognition elements, aptamers may offer opportunities for integration with molecularly imprinted polymers to develop hybrid sensing interfaces. Aptamer-based signal amplification strategies and DNA nanostructures demonstrate how target-induced conformational changes and nucleic acid architectures can be coupled to electrochemical transduction [64,65,66]. In principle, combining sequence-specific aptamer recognition with the physicochemical stability of an imprinted polymer could provide complementary mechanisms for target binding and signal generation. However, polymer incorporation may restrict aptamer folding, reduce binding-site accessibility, or interfere with electron transfer, potentially offsetting the anticipated benefits. Furthermore, additional synthesis and functionalization steps may increase fabrication complexity, reagent consumption, and material waste. Accordingly, improvements in selectivity, operational stability, and analytical sensitivity should not be assumed without direct comparisons against aptamer-only and MIP-only controls. The cited studies support the underlying recognition and transduction concepts but do not, by themselves, establish the performance or sustainability advantages of aptamer–MIP hybrid sensors for antiretroviral drug monitoring.

### 3.2. Aptamers as a Comparator for Green Analytical Chemistry

Evaluating aptamers against the same green-chemistry criteria applied to MIPs clarifies where each recognition strategy’s sustainability burden falls. Aptamer production relies on solid-phase oligonucleotide synthesis, which is chemically demanding: each coupling cycle typically requires anhydrous organic solvents, phosphoramidite reagents, and iterative deprotection and washing steps, and the reagent and solvent burden scales with sequence length and synthesis scale rather than with the number of subsequent assays performed. Once synthesized and validated, however, an aptamer sequence can in principle be manufactured centrally and distributed for use across many sensors without repeating fabrication for each device, in contrast to a MIP layer, which is generally synthesized directly on the individual transducer it will be used with. Aptamers also avoid the extraction-solvent step that MIP fabrication requires to remove the template and open binding cavities, since no analogous extraction step exists in aptamer-based recognition. Neither strategy has a straightforwardly smaller footprint: MIP fabrication concentrates its environmental burden at the point of use, while aptamer production concentrates it upstream, in synthesis. Integration with gold surfaces, self-assembled monolayers, microneedles, and other transducers can support portable sensing [71,72]. As with MIPs, no antiretroviral aptamer study reviewed here reports a standardized greenness metric, so this comparison remains qualitative rather than quantified.

## 4. MIPs as Smart Recognition Components

### 4.1. Design and Fabrication Determinants of MIP Performance

MIPs are attractive for antiretroviral sensing because they provide synthetic recognition sites without relying on enzymes, antibodies, or nucleic acid receptors. This can improve resistance to temperature, pH, and storage conditions and may reduce cold-chain requirements [73,74]. Small antiretroviral molecules are also compatible with cavity-based recognition because their size and functional groups can be represented within a cross-linked polymer network. These benefits do not make MIPs inherently selective or sustainable. Selectivity depends on the chemical complementarity and accessibility of the imprinted sites, while sustainability depends on the monomers, crosslinkers, solvents, extraction steps, electrode materials, reusability, and end-of-life handling used in the complete device.

Understanding where each of these factors is introduced requires tracing the fabrication sequence itself, from initial template association through to the final transducer response. MIP fabrication begins with association of the antiretroviral template with one or more functional monomers, followed by polymerization in the presence of a crosslinking environment. In electrochemical devices, electropolymerization is especially useful because film growth can be controlled through the applied potential, scan range, cycle number, or deposition time [33,53,54,56]. The template is subsequently removed to create binding cavities, and rebinding is measured through a change in current, potential, impedance, or another transducer response. The apparent simplicity of this sequence can obscure several critical variables. Excessively thick films slow mass transfer and can trap template molecules, whereas very thin films may contain too few stable recognition sites. Strong monomer–template interactions can improve the precision of the imprinted cavity but may also make extraction difficult. Harsh extraction solvents can damage the polymer, electrode, or supporting nanomaterials. Accordingly, optimization focuses on balancing imprinting quality, extraction efficiency, rebinding kinetics, nonspecific adsorption, and operational stability rather than maximizing a single electrochemical signal.

For newer integrase strand transfer inhibitors and long-acting antiretroviral agents, imprint design should be guided by the physicochemical properties of the target molecule rather than the direct adaptation of previously successful fabrication protocols. Dolutegravir, bictegravir, cabotegravir, and lenacapavir differ in molecular size, ionization behavior, hydrophobicity, and hydrogen-bonding characteristics, which can influence template–monomer interactions, polymer formation, and subsequent analyte recognition. Functional monomers and polymerization conditions must therefore be selected to balance sufficiently strong template interactions during imprint formation with reversible binding during analyte detection. Solvent composition and crosslinking density can further influence cavity formation, template extraction, and rebinding kinetics. Although thin, surface-accessible imprinted films may reduce diffusion limitations, they can also restrict the number of available binding sites and remain susceptible to nonspecific adsorption and biofouling. Consequently, non-imprinted polymer controls, time-dependent rebinding studies, and matrix-matched interference experiments are essential for establishing whether the measured response reflects selective molecular recognition rather than nonspecific interactions or changes in electrode properties [33,54,73,74].

An additional challenge is the heterogeneity of recognition sites generated during molecular imprinting. Variations in template–monomer interactions and local polymerization conditions can produce binding cavities with different geometries, chemical environments, affinities, and accessibility. Consequently, a strong electrochemical response does not necessarily indicate uniform or highly selective recognition, particularly when structurally related compounds or matrix constituents can interact with lower-affinity binding sites. These effects become especially important at low drug concentrations, where nonspecific adsorption and differences in binding-site occupancy may influence analytical accuracy and reproducibility [33,53,54,55,73]. Binding kinetics introduce a further constraint because analytes must diffuse through the polymer and access recognition cavities before a stable analytical response can be obtained. Deeply embedded sites, dense crosslinked networks, and excessive film thickness may prolong equilibration and reduce the fraction of binding sites accessible within clinically practical measurement times. Importantly, polymer fabrication time and analytical response time should be evaluated separately, since prolonged sensor preparation does not necessarily imply slow analyte detection. Establishing suitability for decentralized antiretroviral monitoring therefore requires reporting incubation time, response kinetics, inter-electrode reproducibility, and performance under the intended sample conditions rather than relying primarily on detection limits.

Surface imprinting and nanoscale molecularly imprinted polymers (nano-MIPs) represent alternative strategies for addressing the mass-transfer limitations and restricted binding-site accessibility associated with conventional bulk-imprinted films. Surface imprinting aims to position recognition cavities near the polymer–solution interface, thereby reducing diffusion distances and potentially improving analyte binding and release kinetics. Nano-MIPs offer a different architecture in which the small particle dimensions can increase the proportion of accessible recognition sites and shorten intraparticle transport pathways. Solid-phase imprinting methods, in which templates are immobilized on a supporting surface during particle synthesis, may also facilitate the selection or enrichment of particles with desirable binding characteristics. However, these potential advantages must be weighed against nanoparticle aggregation, immobilization efficiency, interfacial electron-transfer limitations, and additional fabrication requirements. Although broader studies of MIPs and nanomaterial-based recognition systems provide a foundation for these approaches [73,74], their specific analytical benefits for antiretroviral drug monitoring should not be assumed without experimentally validated comparisons of selectivity, binding kinetics, detection limits, reproducibility, and performance in relevant biological matrices.

These fabrication choices carry direct sustainability consequences and cannot be optimized on analytical performance alone. Template-extraction solvents are frequently the largest single reagent input in an otherwise miniaturized device: acidic, organic, or surfactant-based washes can require repeated large-volume rinsing cycles that offset the sample-volume savings gained from electrochemical miniaturization. Selecting functional monomers and crosslinkers that permit extraction in aqueous or low-hazard solvents, minimizing the number of electropolymerization and washing cycles, and designing films thin enough to extract completely without prolonged solvent exposure are therefore green-chemistry decisions as much as they are analytical ones. Reporting solvent identity, volume, and extraction time alongside detection limits would allow these trade-offs to be evaluated rather than assumed.

**Figure 3:**
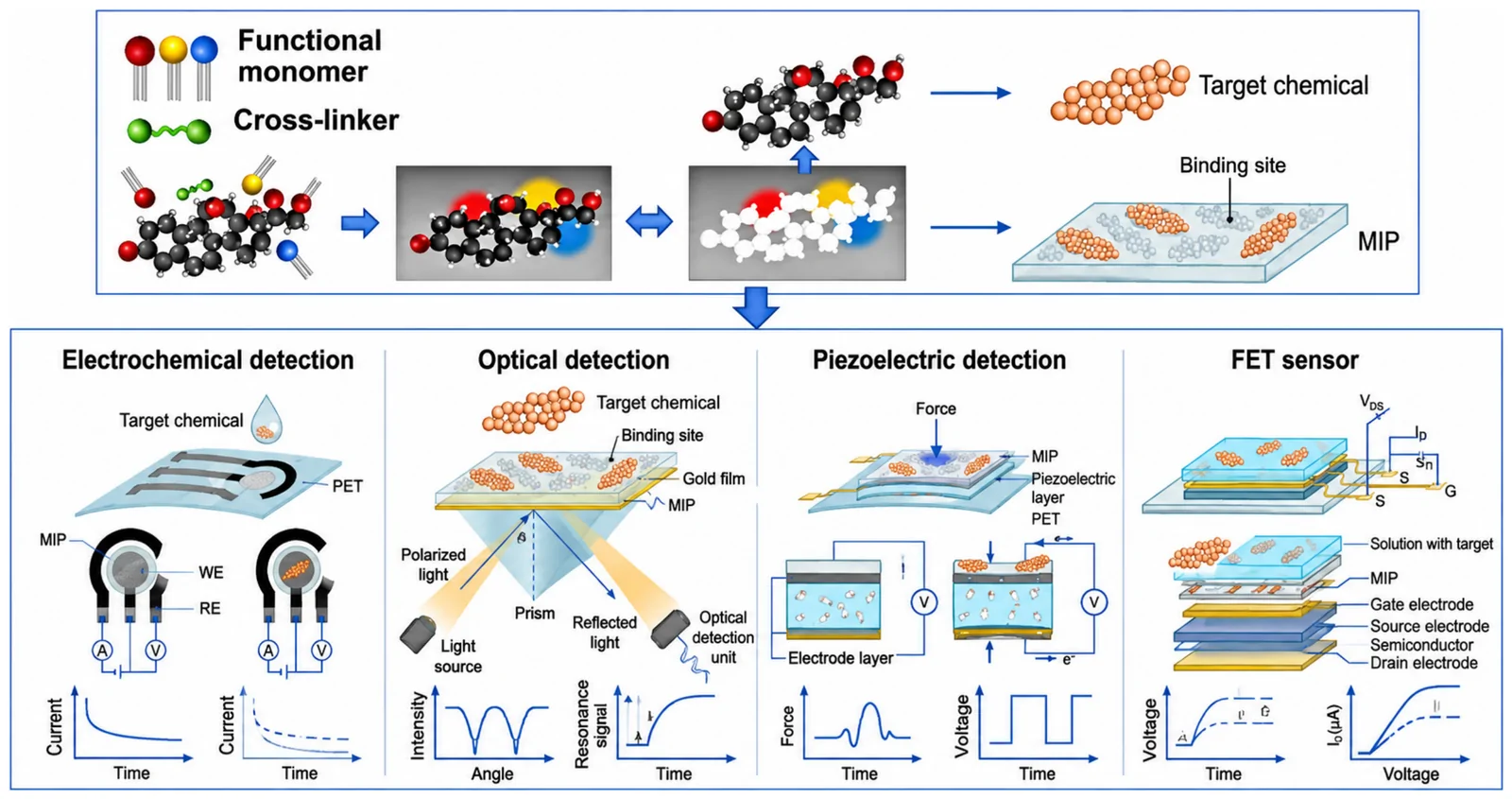
Schematic illustration of molecularly imprinted polymer fabrication and detection mechanisms for sensor types. Adapted from Li et al. (2025) [73].

Figure 3 summarizes this fabrication sequence in general form and situates electrochemical transduction within the broader set of readout strategies available to MIP-based sensors [73]. The functional monomer and cross-linker first assemble around the target chemical to form a pre-polymerization complex; polymerization locks this arrangement into place, and template removal leaves behind binding sites complementary in shape and chemistry to the original target, which the MIP can then rebind during an assay. This same imprinted layer can, in principle, be coupled to several different transducer types, and Figure 3 illustrates four: electrochemical detection, where rebinding alters the current or voltage measured at a working electrode; optical detection, where rebinding shifts the resonance signal of reflected polarized light; piezoelectric detection, where rebinding changes the mechanical resonance of a piezoelectric layer under applied force; and field-effect transistor detection, where rebinding at the gate modulates the current passing between source and drain. The antiretroviral MIP platforms discussed here rely almost exclusively on the electrochemical route shown at left, reflecting its compatibility with portable, low-power instrumentation; the optical, piezoelectric, and FET formats shown alongside it illustrate alternative transduction strategies that have seen comparatively little application to antiretroviral targets specifically, representing a direction for future work rather than a current body of evidence.

These general fabrication principles are illustrated by the tenofovir, emtricitabine, and nevirapine platforms already compared in Section 2.2, where increasingly elaborate composite architectures combining MWCNTs, platinum nanoparticles, graphitic carbon nitride, MXenes, and metal–organic frameworks did not consistently translate into proportionally stronger clinical evidence. The growing emphasis on polymer architecture and binding-site accessibility reflects broader advances in molecular imprinting, which has expanded from predominantly small-molecule recognition to applications involving proteins, nucleic acids, and other biomolecular targets [28,75]. These developments have encouraged greater control over polymer morphology, recognition-site distribution, and integration with nanostructured transducers. However, successful imprinting of larger biomolecules does not necessarily establish the suitability of similar fabrication strategies for antiretroviral drugs, whose molecular structures, physicochemical properties, and clinically relevant concentration ranges present distinct analytical requirements. For antiretroviral monitoring, the value of these advances therefore lies not in directly transferring previously reported imprinting protocols, but in identifying fabrication principles that improve binding-site accessibility, selective recognition, and reproducible electrochemical transduction without introducing unnecessary processing complexity.

Beyond these drug targets, fabrication strategy itself is evolving toward simpler, more scalable formats. Laser-scribed graphene and gold nanostructures offer a potentially scalable route to integrating MIPs with disposable electrochemical transducers. Their value for green analytical chemistry depends on whether fabrication reduces energy, solvent, and material consumption relative to more complex multilayer composites. Reusability can reduce per-test waste, but only when regeneration does not require large solvent volumes or cause progressive loss of binding performance. Few studies currently report these factors alongside detection limits and recovery data, which is what makes this trade-off difficult to evaluate at present.

### 4.2. MIPs as Smart Biocomponents for Green Analytical Chemistry

MIPs can support green analytical chemistry by replacing fragile biological receptors with stable synthetic recognition layers, reducing the need for refrigerated transport and storage, and enabling miniaturized measurements with microliter sample volumes. Electrochemical formats can also reduce energy demand relative to centralized chromatographic instrumentation and can support same-visit testing with portable potentiostats. These advantages are conditional rather than automatic. A MIP sensor that requires multiple nanoparticle syntheses, noble metals, toxic monomers, large solvent volumes, or single-use multilayer substrates may shift environmental burdens from the analytical laboratory to sensor fabrication and disposal.

The most credible green advantages arise from simple electropolymerized films, screen-printed or laser-patterned electrodes, aqueous or low-hazard fabrication media, minimal template-extraction volumes, and validated regeneration. Paper and carbon-based supports may reduce material demand, but their sustainability depends jointly on conductive inks, binders, nanomaterials, and packaging. Reporting recognized green analytical metrics, or at minimum transparent data on solvent type and volume, sample consumption, energy requirements, number of reusable cycles, and waste generated per analysis, is recommended for future studies.

A rigorous assessment of MIP-based antiretroviral sensors as green analytical tools requires more than qualitative claims of portability or miniaturization. Established greenness assessment frameworks, including the Analytical GREEnness (AGREE) metric, the Green Analytical Procedure Index (GAPI), and the Analytical Eco-Scale, provide standardized, semi-quantitative scoring of sample preparation, reagent hazard, energy consumption, and waste generation. None of the antiretroviral MIP studies summarized here report scores from any of these frameworks. This is not primarily a reporting gap. Of the 13 antiretroviral MIP and comparator studies examined in this review, 10 report the solvent identity and volume, extraction or preparation time, and number of steps needed to retrospectively reconstruct AGREEprep or GAPI scores. The remaining three omit at least one required parameter, most commonly solvent volume, drying time, or details of upstream composite synthesis from the main manuscript. The absence of greenness scores in this literature therefore reflects a failure to apply available frameworks to already-available data, rather than a shortfall in the underlying methodological detail. Retrospectively applying AGREEprep or GAPI to these published protocols, or reporting them prospectively during future method development, would let the field move from qualitative sustainability language to reproducible, comparable greenness metrics with comparatively little additional effort. Until such assessments become standard practice, claims that a given MIP platform is green or sustainable should be treated as provisional rather than established. Table 2 summarizes this assessment across all 13 studies, evaluated against the four parameters needed to reconstruct an AGREEprep or GAPI score retrospectively.

### 4.3. Critical Comparison with Alternative Recognition Strategies

Compared with direct electrooxidation, MIPs generally provide stronger molecular recognition but introduce additional fabrication and extraction steps. Compared with aptamers, MIPs offer greater resistance to temperature and storage conditions, but aptamers may provide more uniform binding chemistry when a validated sequence is available. Compared with antibodies, MIPs can reduce cost and cold-chain dependence, but antibodies retain stronger clinical precedent. Therefore, MIPs are most compelling when they deliver a measurable advantage in stability, reusability, or decentralized operation without requiring a level of material complexity that undermines manufacturability or sustainability.

A rigorous comparison of these strategies requires distinguishing molecular recognition from signal transduction. Recognition determines the extent to which a sensing interface selectively interacts with the target analyte, whereas transduction determines how that interaction is converted into a measurable response. Molecular imprinting primarily addresses the recognition component by creating polymeric binding sites complementary to a target molecule, but its analytical performance also depends on the properties of the underlying transducer and the efficiency of signal coupling. Consequently, MIP-based recognition can be integrated with electrochemical, optical, or other compatible transduction mechanisms. Improvements in signal amplitude or detection limits should therefore not be attributed solely to molecular imprinting without separating the contributions of recognition chemistry, electrode or device architecture, and signal amplification.

Electrochemical transduction is particularly attractive for decentralized antiretroviral monitoring because voltammetric and impedimetric measurements can be implemented using compact instrumentation and small sample volumes, although biofouling, matrix interference, and electrode-to-electrode variability remain important limitations [33,53,54]. Optical approaches can provide complementary detection mechanisms without requiring direct analyte electroactivity, but their suitability for point-of-care deployment depends on optical alignment, instrumentation requirements, and device integration. Magnetic detection represents a different analytical principle and requires careful distinction between detecting a magnetic label and selectively recognizing a drug molecule. For example, Barrera et al. [76] demonstrated magnetoimpedance-based detection of magnetic iron oxide particles, while Melnikov et al. [77] investigated magnetic-particle detection in a blood-vessel model. Although these studies illustrate the capabilities of magnetic transduction, they do not establish selective recognition or quantification of antiretroviral drugs. Their reported detection performance therefore cannot be directly compared with the detection limits of MIP-based antiretroviral sensors.

The practical advantages of electrochemical MIP-based sensors therefore depend on demonstrating selective recognition, reproducible calibration, acceptable matrix interference, operational stability, and compatibility with low-cost, portable instrumentation. Alternative transduction approaches may offer advantages for particular applications, but no single recognition–transduction combination can be considered universally superior in sensitivity, selectivity, sustainability, or clinical readiness. These outcomes depend on the complete sensing architecture, including recognition chemistry, signal generation, fabrication requirements, instrumentation, sample preparation, and waste management. As discussed by Erdem et al. [75], molecularly imprinted interfaces can support biomolecular sensing, although recognition performance and transducer response should be evaluated as distinct contributions. Meaningful comparisons for antiretroviral monitoring therefore require benchmarking against the same target analyte, biological matrix, concentration range, and reference method, using consistent metrics such as detection limit, analytical recovery, selectivity, response time, reproducibility, stability, and environmental burden. Without such comparisons, apparent differences in performance may reflect experimental conditions or transducer design rather than inherent advantages of the recognition strategy.

Table 3 summarizes these trade-offs across all four strategies, including the green-analytical-chemistry implications. Each strategy’s principal limitation points to a distinct barrier: selectivity for direct electrooxidation, selection and immobilization for aptamers, biological reagent burden for antibodies, and fabrication-driven variability for MIPs, so no single strategy is positioned to dominate the others across every criterion in the table simultaneously.

The principal strengths of MIP-based antiretroviral sensors include chemical stability, compatibility with miniaturized transducers, potential regeneration, and the ability to tailor recognition toward small drug molecules. However, heterogeneous binding sites, nonspecific adsorption, incomplete template removal, slow rebinding in densely crosslinked films, and batch-to-batch variability remain important limitations. These challenges can become more pronounced in complex biological matrices, where proteins, salts, metabolites, and coadministered drugs may interfere with analyte recognition or alter the transducer response. Residual template leakage may further compromise analytical accuracy, particularly at low target concentrations. Consequently, lower detection limits alone do not establish clinical suitability. Meaningful validation requires selectivity testing against relevant antiretroviral drugs and endogenous interferents, reproducibility across independently fabricated batches, storage and regeneration stability, matrix-matched calibration, and comparison with validated reference methods such as LC-MS/MS. From a sustainability perspective, sample volume, solvent and reagent consumption, energy requirements, sensor reusability, and disposable material burden must also be evaluated. Collectively, these considerations highlight the need for more comprehensive analytical and environmental validation than is typically conveyed by detection limits and recovery measurements alone.

The emtricitabine LIG/Prussian Blue/MIP sensor provides a useful example of progress toward more rigorous analytical validation [33]. By incorporating a non-imprinted polymer control, evaluating interference from structurally related antiretroviral drugs and common electroactive species, and testing performance in synthetic urine and protein-enriched synthetic urine, the study addressed several limitations frequently overlooked in early-stage MIP sensor development. Nevertheless, these experiments do not fully establish performance in authentic human biological samples, where variable matrix composition, nonspecific adsorption, and potential biofouling may affect recognition and signal reproducibility. The reported limitations in regeneration and longer-term sensor variability further illustrate the distinction between demonstrating analytical feasibility and establishing a clinically deployable sensing platform. This example underscores the importance of progressing from controlled laboratory validation to testing in authentic biological matrices, independently fabricated sensor batches, and direct comparisons with established reference methods.

The emtricitabine LIG/Prussian Blue/MIP platform discussed in Section 2.2 illustrates both the progress and remaining challenges in validating MIP-based antiretroviral sensors [33]. Although its non-imprinted controls, interference studies, and evaluation in synthetic and protein-enriched urine provide stronger evidence of analytical selectivity and matrix compatibility than buffer-only testing, these results do not establish reliability in authentic human samples. Furthermore, the reported limitations in regeneration and longer-term reproducibility highlight the need for systematic evaluation of sensor durability and batch-to-batch performance. This distinction between analytical feasibility and clinical readiness reinforces the importance of standardized validation protocols, reference-method comparisons, and comprehensive assessment of the environmental costs associated with sensor fabrication and use.

## 5. Point-of-Care Testing and Challenges Limiting Widespread Implementation

### 5.1. Resistance Testing, Clinical Actionability, and Same-Visit Testing

Clinically matching a sensor to its intended use also means being precise about what that sensor does and does not measure. HIV diagnosis, viral-load monitoring, resistance testing, and antiretroviral drug sensing address different clinical questions. Drug sensing does not replace diagnosis or viral-load testing; its value is to provide objective evidence of recent or cumulative drug exposure that may help explain adherence or treatment failure [70,71,72,73,74,75,76,77,78,79,80,81,82]. Point-of-care drug sensing may complement counseling and clinical laboratory testing when results are linked to validated exposure windows, but claims that a device detects resistance or predicts treatment failure are not supported unless the assay directly measures the relevant viral or pharmacologic endpoint. Drug-resistance testing evaluates viral mutations, whereas antiretroviral sensing evaluates drug exposure; the two approaches are complementary but analytically distinct [6,81]. This review therefore treats resistance testing only as contextual background and does not evaluate it as an MIP drug-sensing application.

Whether a MIP-based antiretroviral sensor can move beyond the laboratory depends on two linked questions: what makes a result clinically actionable at the point of care, and what practical barriers stand between a validated platform and real-world deployment. Clinical actionability requires a result within the intended care encounter, minimal sample preparation, limited user training, and an output linked to a validated adherence or therapeutic threshold. Low cost and portability are important, but they do not compensate for poor reproducibility, uncertain calibration, or lack of reference-method agreement [82]. Urine tenofovir assays illustrate the value of same-visit adherence testing because recent exposure can be assessed without centralized laboratory equipment [6]. For MIP platforms, the key question is whether comparable speed and simplicity can be achieved while maintaining selectivity in authentic urine and avoiding solvent-intensive regeneration. The clinical value of rapid drug-level testing lies in supporting timely, nonjudgmental adherence counseling and treatment decisions [83,84]. Sensor results are therefore best interpreted within the pharmacologic window represented by the matrix, not as a complete measure of long-term adherence.

Same-visit testing is not a single, uniform target: the specific question a sensor needs to answer, and the environment in which it must answer that question, both shape what “clinically actionable” requires in practice. PrEP monitoring and treatment monitoring address different clinical questions. PrEP testing often emphasizes recent exposure sufficient for protection, whereas treatment monitoring may require longer-term assessment of adherence, pharmacokinetics, toxicity, and resistance risk [85]. MIP sensor design therefore depends on the intended drug, matrix, concentration range, and decision threshold. Deployment setting imposes a further, independent set of constraints. Decentralized deployment in low- and middle-income settings requires more than a portable reader: devices must tolerate variable power and temperature, use dependable supply chains, minimize consumables, and support training by local healthcare workers [6,86,87,88,89]. Designs that depend on fragile reagents, multiple extraction solutions, or complex multilayer materials may be poorly suited to these environments.

### 5.2. Clinical Translation, Regulatory, and Commercialization Barriers

Delivering a result that is both clinically scoped correctly and analytically trustworthy is a separate challenge from the scope question itself. Analytical challenges arise from the low concentrations of antiretroviral drugs and the complexity of clinical matrices. Proteins, salts, metabolites, and structurally related drugs can produce nonspecific adsorption or cross-reactivity. For MIPs, assessing performance requires non-imprinted controls, relevant interferents, independently fabricated batches, and matrix-matched calibration; results obtained only in buffer or synthetic urine are insufficient to establish clinical selectivity. Clinical translation requires direct comparison with LC-MS/MS using authentic specimens that represent the intended population and sampling matrix. Without this evidence, even analytically sensitive devices remain proof-of-concept tools.

Incomplete template removal represents an additional challenge for the clinical translation of MIP-based antiretroviral sensors. Residual template molecules may gradually leach from imprinted cavities during subsequent measurements, potentially contributing to background signals or positively biased estimates of drug concentration, depending on the sensing mechanism. Such interference is particularly concerning when measuring low antiretroviral concentrations, where even small contributions from residual template may compromise the distinction between sensor-derived contamination and drug originating from the patient sample. Inaccurate concentration measurements could subsequently lead to incorrect interpretations of drug exposure or adherence. Slow analyte rebinding and equilibration may further prolong measurement times, limiting the suitability of these platforms for same-visit clinical decision-making. Accordingly, preclinical analytical validation should include post-extraction blank measurements, assessment of template leakage during repeated assays, characterization of binding and response kinetics, and evaluation of recovery and reproducibility across relevant concentration ranges. These studies should be complemented by comparisons with validated reference methods, such as LC-MS/MS, using authentic biological samples to establish analytical accuracy and suitability for the intended clinical application [33,53,54,57,72]. Together, these measurements are necessary to distinguish genuine analyte recognition from residual-template interference and to determine whether the sensor can provide accurate results within clinically practical testing times.

Even a platform that clears analytical and clinical validation still faces a distinct set of barriers before it reaches routine use, spanning manufacturing, regulatory approval, and commercial viability. Device engineering must address shelf life, sensor-to-sensor variation, calibration transfer, packaging, fluid handling, and user operation. Complex hybrid MIP electrodes can be difficult to manufacture reproducibly because small changes in film thickness, nanomaterial loading, extraction, temperature, or curing conditions alter the response. Designs intended for scale-up therefore minimize the number of material layers and fabrication steps and use process controls that can be transferred from laboratory preparation to batch manufacturing.

Manufacturing reproducibility is, in turn, a prerequisite for regulatory approval rather than a separate track running in parallel. Regulatory implementation requires predefined analytical acceptance criteria, stability testing, quality controls, and a clear intended use. Adoption will also depend on cost, reimbursement, supply chains, user training, data privacy, and confidence among patients and clinicians. Portability or low sample volume alone is too thin a basis for a sustainability claim; credible claims require evidence concerning material hazards, energy use, reagent consumption, reusability, and waste across fabrication, operation, and disposal. Clearing regulatory requirements still does not guarantee commercial adoption. Commercialization depends on material cost, process reproducibility, intellectual property, clinical evidence, market size, reimbursement, and manufacturing partnerships [90,91,92,93,94]. The strongest commercial candidates are unlikely to be the most analytically elaborate devices; they are more likely to be platforms that provide sufficient performance with fewer material layers, fewer extraction steps, stable supply chains, and a clearly defined clinical use.

Translating these comparisons into practice, Table 4 summarizes ranked recommendations for recognition-strategy selection across four antiretroviral monitoring scenarios discussed throughout this review. These recommendations reflect the qualitative comparisons developed in Section 2, Section 3, Section 4 and Section 5 rather than quantified greenness scores, since the underlying AGREEprep or GAPI data are not yet available for the reviewed studies.

## 6. Emerging Directions for Sustainable and Clinically Relevant MIP Biosensors

The preceding sections evaluated MIP-based antiretroviral sensors largely as they exist today: single-analyte, clinic-based, and validated primarily in prepared or synthetic matrices. Four directions currently under exploration would extend this work toward continuous, multi-target, and data-integrated monitoring, though each raises questions that current MIP designs are not yet positioned to answer. Wearable and minimally invasive platforms could extend antiretroviral monitoring beyond episodic clinic visits, particularly through interstitial fluid or sweat sampling [95]. For MIPs, the unresolved questions are whether binding and regeneration remain stable during repeated use, whether matrix concentrations are sufficient, and whether the added electronics and packaging preserve the claimed sustainability benefit.

Drug-specific sensor development is particularly important for integrase strand transfer inhibitors and long-acting antiretroviral agents, whose pharmacokinetic profiles create different monitoring requirements. Dolutegravir illustrates the importance of understanding changes in plasma and urinary drug concentrations following dosing [16], while long-acting cabotegravir and lenacapavir introduce additional considerations associated with prolonged exposure and extended dosing intervals [14,15,17]. For these agents, MIP-based sensors must be evaluated against concentration profiles relevant to the intended monitoring objective, including recent dosing, sustained exposure, or declining concentrations between administrations. Analytical sensitivity alone is insufficient to establish clinical utility; the sensor must also demonstrate appropriate quantification ranges, selectivity, matrix compatibility, and reproducibility across clinically meaningful exposure conditions. Accordingly, future development should prioritize drug-specific analytical validation linked to defined clinical questions rather than treating low detection limits or broad linear ranges as independent indicators of monitoring readiness.

Transducer selection depends on electrochemical behavior/characterization of the drug. Direct voltammetric detection (e.g., cyclic voltammetry, square wave voltammetry) would need a distinct anodic response within the appropriate potential window. If said response is not achieved, then an impedance-based evaluation would be utilized, or a coupling with a film and redox mediator, to procure an indirect measure of rebinding [33,53,54]. Potential concerns could come in the form of matrix biofouling or hydrophobic adsorption, typically resulting from low concentrations or measurement times that are ultimately too short. Any proposed designs must withstand scrutiny through variables such as response time, potential drift, selectivity/interference, sensor variability, and evaluation against established detection methods. Research exploration in this direction with the discussed antiretroviral drugs could produce novel results.

Porous framework materials represent another potential direction for advancing MIP-based antiretroviral sensors beyond conventional metal and carbon nanostructures. The nickel–copper metal–organic framework (MOF) composite reported for nevirapine detection [56] illustrates the application of framework-based materials in electrochemical antiretroviral analysis, although it does not establish molecularly imprinted recognition or improved binding-site uniformity. Covalent organic frameworks (COFs) offer a conceptually attractive alternative because their tunable pore structures and chemical functionality may support controlled polymer integration and accessible recognition interfaces. However, the analytical advantages of MOF- or COF-assisted imprinting would depend on pore accessibility, analyte transport, framework stability during template extraction, fabrication reproducibility, and compatibility with complex biological matrices. Additional framework synthesis and modification steps must also be justified by measurable improvements in selectivity, response kinetics, or analytical reliability relative to their material and solvent requirements. In the absence of validated COF-based MIP examples for antiretroviral monitoring in the literature considered here, these architectures should be regarded as prospective research opportunities rather than established solutions.

Magnetic-assisted molecular imprinting represents a further exploratory strategy for improving sample preparation and recognition-interface integration. Magnetic particles functionalized with imprinted polymers could potentially support selective analyte enrichment, magnetic separation, or controlled positioning of recognition materials near an electrochemical transducer. Magnetic readout is also possible in appropriately designed systems, although magnetic-particle detection alone does not establish molecular selectivity. The magnetoimpedance studies reported by Barrera et al. [77] and Melnikov et al. [78] illustrate magnetic-particle detection capabilities but do not demonstrate MIP-based recognition or quantification of antiretroviral drugs. Accordingly, the proposed benefits of magnetic-assisted MIP systems would require validation using imprinted and non-imprinted controls, relevant antiretroviral interferents, recovery studies, and assessment of particle loss or aggregation. Their practical value would further depend on whether improved analyte enrichment or matrix tolerance justifies the additional materials, separation steps, instrumentation, and waste associated with magnetic integration.

Continuous monitoring raises the question of what a wearable platform needs to measure over time; multiplexing raises a parallel question of how many targets a single platform can measure at once. Multiplexed platforms may support simultaneous measurement of several antiretroviral drugs or combined drug and biomarker panels [96]. Molecular imprinting could contribute through spatially separated recognition layers, but cross-reactivity, calibration complexity, material use, and signal deconvolution will increase accordingly. Multiplexing is therefore justified only when it provides a clear clinical advantage over single-analyte testing, rather than pursued as a default design goal.

A third direction is defined not by platform architecture but by a shift in the pharmacokinetics being monitored. Long-acting injectable therapies create a need to identify unusually low or prolonged drug exposure between scheduled doses [17,97]. Future MIP sensors therefore need to target clinically relevant concentration ranges for cabotegravir, lenacapavir, and related agents and to be evaluated against the pharmacokinetic questions these therapies create, rather than adapting tenofovir designs without target-specific optimization. This distinction matters because a sensor optimized for daily-dosing tenofovir concentrations is not automatically suited to the sustained, low-level exposure windows characteristic of long-acting agents.

The first three directions each generate more complex or more continuous data than current single-timepoint measurements; interpreting that data is itself an emerging challenge. Data analytics may help interpret longitudinal sensor measurements, identify unusual exposure patterns, and communicate trends to clinicians [98,99]. These tools cannot compensate for poorly validated sensor data. Algorithm development should therefore follow, not precede, robust analytical validation, while also addressing privacy, bias, explainability, and data security.

Across all four directions, the same constraint recurs: each adds complexity, whether in materials, calibration, target scope, or data volume, that must be justified against a clearly defined clinical need rather than pursued as a demonstration of technical capability. Wearable formats, multiplexed panels, long-acting-agent targeting, and analytics integration are complementary rather than competing priorities, but none of them substitutes for the fabrication reproducibility, matrix-matched validation, and reference-method agreement established as prerequisites throughout this review. Sustainable, clinically relevant MIP biosensing will depend on extending the field in these directions without abandoning that evidentiary standard.

## 7. Conclusions

MIPs provide a credible route to stable and selective antiretroviral drug sensing because they replace fragile biological receptors with synthetic recognition cavities that can be integrated with electrochemical and optical transducers. The reviewed studies demonstrate nanomolar detection for selected drugs, particularly tenofovir and nevirapine, but they do not establish that increasingly complex hybrid architectures are the most clinically useful or environmentally responsible. Analytical gains are often accompanied by additional material layers, expensive modifiers, solvent-intensive extraction, and greater risk of batch variation. The central challenge is therefore not further reduction of detection limits. It is demonstrating reproducible recognition in authentic clinical matrices while maintaining simple processing, scalable manufacturing, clinically interpretable outputs, and responsible material use. Future studies should compare MIPs with non-imprinted controls and validated reference methods, report cross-reactivity and independent-batch reproducibility, evaluate regeneration and storage stability, and quantify sample, solvent, reagent, energy, and waste requirements using recognized greenness metrics. Molecular imprinting will have the greatest value as a smart biocomponent for green analytical chemistry when its robustness and reusability produce measurable reductions in resource demand without compromising clinical reliability.

## Figures and Tables

**Table 1:** Comparison of representative electrochemical biosensors for antiretroviral drug monitoring.

Sensor|Analyte	Method	LR	LOD	Sample	Ease of Fabrication	Ref.
LIG/PB/MIP| FTC	SWV	10 nM–200 µM	5 nM	Synthetic urine, synthetic urine + protein	Moderate	[33]
SAM/MCH|TFV	FET	1–100 nM	1.2 nM	Serum	Moderate	[52]
LSG/AuNSp/MIP| TFV	SWV	0.1–200 µM	3 nM	Synthetic urine, synthetic urine + serum	Moderate	[53]
MIP/Pt@g-C_3_N_4_/F-MWCNT| TFV	DPV, EIS	0.005–0.69 µM	3 nM	Biological fluids, water samples	Complex	[54]
MIP/ErGO/GCE| NVP	DPV	0.005–400 µM	2 nM	Pharmaceuticals, serum	Moderate	[55]
NiCu-MOF/Ti_3_C_2_/MWCNTs/GCE| NVP	DPV	0.01–50 µM	1.2 nM	Biological fluids	Complex	[56]
Ni-CoS-GQD| TFV	DPV	10–60 µM	56.18 µg/mL	N/A	Moderate	[57]
GO/GCE| TFV	SWV	0.3–30 µM	48.6 nM	Pharmaceuticals, urine	Moderate	[58]
Hg(Ag)FE| TFV		0.5–7 µM	13.5 µM			
ErGO-Pt/Nafion/EPPG| EFV	SWV	0.05–150 µM	1.8 nM	Pharmaceuticals, urine, plasma	Moderate	[59]
GCE/NiO-ZrO_2_| EFV	CV	0.01–10 mM	1.4 nM	Pharmaceuticals, urine	Moderate	[60]
PGE| EFV	DPASV	0.05–8.11 µM	13.8 nM	Pharmaceuticals	Simple	[61]
BZK/AgNPs/GCE| TFV	SWCAdSV	0.06–1 µM	2.39 nM	Pharmaceuticals	Moderate	[62]

Abbreviations: LSG—laser scribed graphene, AuNSp—gold spiky nanostructure, MIP—molecularly imprinted polymer, TFV—tenofovir, SWV—square wave voltammetry, SAM—self-assembled monolayer, MCH—6 mercapto-1 hexanol, FET—field effect transistor, Pt—platinum, g-C_3_N_4_—graphitic carbon nitride, F-MWCNT—functionalized multi-walled carbon nanotubes, DPV—differential pulse voltammetry, EIS—electrochemical impedance spectroscopy, Ni-CoS-GQD—nickel-cobalt sulfide decorated graphene quantum dots, BZK—benzalkonium chloride, AgNPs—silver nanoparticles, GCE—glassy carbon electrode, SWCAdSV—square-wave cathodic adsorptive stripping voltammetry, GO—graphene oxide, Hg(Ag)FE—cyclic renewable mercury film silver-based electrode, NiCu-MOF—nickel & copper-based metal–organic framework, Ti_3_C_2_—2 dimensional titanium carbide (MXene), NVP—nevirapine, ErGO—electrochemically reduced graphene oxide, EPPG—edge plane pyrolytic graphite, NiO-ZrO_2_—nickel oxide-zirconium dioxide, PGE—pencil graphite electrode, DPASV—differential pulse anodic stripping voltammetry. Notes: Ease of Fabrication: Simple = fewer steps, commonly available materials; Moderate = more steps or specific conditions; Complex = requires specialized synthesis or infrastructure.

**Table 2:** Reconstructability of AGREEprep or GAPI scores from reported studies.

Study	Solvent Identity	Solvent Volume	Extraction/Prep Time	Step Count	Reconstructable
[33]	✓	✓	✓	✓	Yes
[51]	✓	✓	N/A	✓	Yes
[52]	✓	✓	✓	✓	Yes
[53]	✓	✓	✓	✓	Yes
[54]	Composite synthesis in ESI	Not in main text	✓	Partial	Partial
[55]	✓	✓	✓	✓	Yes
[56]	✓	Deposition volume not reported	✓	✓	Partial
[57]	✓	✓	✓	✓	Yes
[58]	✓	✓	N/A	✓	Yes
[59]	✓	✓	✓	✓	Yes
[60]	✓	Drying time not reported	Partial	Sparse (3 steps)	Partial
[61]	✓	✓	N/A	✓	Yes
[62]	✓	✓	N/A	✓	Yes

N/A indicates no chemical extraction step exists for that platform (a direct nanomaterial-modified electrode rather than a molecularly imprinted polymer), not that the parameter went unreported. Partial indicates at least one parameter was not resolvable from the available text.

**Table 3:** Critical comparison of recognition strategies for sustainable antiretroviral drug sensing.

Recognition Strategy	Principal Strength	Principal Limitation	Green Analytical Implication
Direct electrooxidation	Rapid and simple	Limited molecular selectivity in complex matrices	Low reagent use, but added modifiers may be required
Aptamer	Programmable affinity	Selection and immobilization challenges for small drugs	Low sample use, with biological reagent and storage demands
Antibody	Strong clinical precedent	Cost, denaturation, and cold-chain dependence	Higher biological reagent and storage burden
MIP	Stable synthetic recognition and potential reuse	Site heterogeneity, template removal, and batch variation	Strong potential when fabrication, extraction, and disposal are simplified

**Table 4:** Ranked recommendations for recognition-strategy selection by ART monitoring scenario.

Analytical Goal	First Choice	Second Choice	Avoid
Rapid, same-visit adherence screening (e.g., urine tenofovir, PrEP context)	Direct electrooxidation [58,62] or a single-layer MIP with minimal fabrication steps, where selectivity requirements are modest [53]	Aptamer-based sensor where a validated sequence exists for the target drug [52]	Multilayer MIP nanocomposites requiring extensive extraction before same-visit results are needed [54]
Quantitative, clinically validated drug-level monitoring (TDM)	MIP platform validated with matrix-matched calibration and direct agreement against LC-MS/MS	Antibody-based immunosensor where clinical precedent is required	Any platform validated only in buffer or synthetic urine without authentic-specimen comparison [33,53]
Long-acting injectable (cabotegravir, lenacapavir) monitoring *	MIP or aptamer platform specifically optimized for the sustained, low-concentration exposure windows of long-acting agents	Adapted tenofovir-designed platform, only after re-validation against the relevant concentration range	Unmodified tenofovir-designed platforms applied to long-acting agents without target-specific optimization
Decentralized or resource-limited deployment	Simplest architecture meeting minimum selectivity requirements: fewest fabrication and extraction steps, lowest solvent burden	Reusable MIP platform with validated regeneration, where consumable supply chains are constrained [33]	Complex hybrid nanomaterial composites requiring noble metals, cold-chain reagents, or multistep extraction [54,56]

* No reviewed study currently meets the criteria described for long-acting-agent monitoring; the gap itself motivates this recommendation.

## Data Availability

Dataset available on request from the authors.
